# Critical conditions for escape of a high-speed fullerene from a BNC nanobeam after collision

**DOI:** 10.1038/s41598-017-18789-7

**Published:** 2018-01-17

**Authors:** Kun Cai, Li-Kui Yang, Jiao Shi, Qing-Hua Qin

**Affiliations:** 1 College of Water Resources and Architectural Engineering, Northwest A&F University, Yangling, 712100 China; 2 Research School of Engineering, the Australian National University, ACT, 2601 Australia

## Abstract

For a resonator-based nano-balance, the capability of capturing a nanoparticle is essential for it to measure the mass of the particle. In the present study, a clamped-clamped nanobeam from a Boron-Nitride and Carbon (BNC) nanotube acts as the nano-balance, and a fullerene, e.g., C_60_, is chosen as the particle, and the capturing capability is quantitatively estimated by the minimal escape velocity (MEV) of the fullerene from the nanobeam after collision. When centrally colliding with the nanobeam, the escape of fullerene depends on both incidence of fullerene and temperature of the system. When the colliding in the Boron-Nitride (BN) area of the beam surface, the nanoball escapes easier than that at the carbon area. The MEV of the nanoball is lower at higher temperature. As the nanoball sometimes slides for a few pica-seconds on the beam surface before being bounced out, the nanoball can escape only when the beam surface can provide the nanoball enough kinetic energy to overcome the van der Waals interaction between them. The capturing capability of the nano-balance can, thus, be improved by reducing the initial kinetic energy of the system.

## Introduction

Like allotropes of carbon, e.g., 1D nanotube^1,2^, 2D nanosheet and 3D diamond, the hybrid nanostructures of boron and nitrogen also have some specific configurations. For example, hexagonal BN sheet looks like graphene^3,4^, and each boron or nitrogen atom is covalently bonded with three other atoms and the sp^2^ B-N bonds are coplanar. An inner carbon atom in graphene has a delocalized electron, which results in the electrical conductivity of graphene. However, neither boron nor nitrogen atom in BN sheet has delocalized electron. Hence, BN sheet is an electrical insulator with band gap of ~5 eV^5^. But the B-N bonding has a partially ionic character which results in strong attraction between adjacent BN sheets. On the other hand, the mechanical^6^ and thermal properties^7–9^ of BN sheet are as good as or better than those of graphene. A carbon nanotube (CNT), which has been widely used in nanodevices^10–15^, can be considered as a nanostructure formed by winding a graphene ribbon along certain direction, which has been numerically verified on both carbon^16^ and other 2D materials, e.g., black phosphorene^17–22^. Similarly, BN nanotube (BNNT)^23,24^, has been synthesized for years^23,25–27^. Due to the properties of the covalent bonds in shell, a BNNT also shows an electrical insulator, which is independent of its chirality. For a BNNT and a CNT with same chirality, the Young’s modulus of a BNNT is lower, but its thermal stability is higher. Hence, BNNTs have wide applications in engineering structures and devices, e.g., in nano-sensor^28–30^, high-temperature ceramics^31^, etc.

Inspired by similar mechanical and thermal properties but different electrical properties between BN nanotube and CNT, people believes that a hybrid nanotube constructed from boron, nitrogen and carbon, i.e., BNC nanotubes, may have some special properties different from either individual CNTs or BNNTs. Interestingly, such hybrid nanotubes have been synthesized. For example, Enouz *et al*.^32^ presented a way to fabricate the BNC nanotubes using a continuous CO_2_ laser vaporization reactor. On the other hand, An and Turner^33^ used density functional theory to investigate the electrical property of heteronanotubes. Zhang and Meguid^34^ investigated the stability of BNC nanotube under uniaxial compression.

Unlike other nanodevices, including nano-oscillator^35–37^ and nanomotor^38–43^, the nanobalance was proposed^44^ for measuring the mass of a nanoparticle. The mechanism of the measuring method is to find the relationship between the mass of an external nanoparticle and the variation of the resonant frequency of the resonator-type balance^28,30,45–47^. With the continuous development, the sensitivity of the nano-resonator has been significantly improved in recent years. Now, it can measure the mass of a single molecular or a cell^28,48^. For example, Sazonova *et al*.^45^ investigated the electrical actuation and detection of the vibration modes of clamped-clamped CNT-based oscillators, whose resonance frequency can be widely tuned and used to measure very small force. In the investigation of the vibration of a CNT-based resonator, Garcia-Sanchez *et al*.^46^ used a tip to attract nanotube for reducing the resonant frequency. In 2008, Knobel^29^ developed a method to test the mass of atoms using a CNT. Chiu *et al*.^49^ designed a carbon nanotube-based resonator to test the mass at atomic-scale. Gil-Santos *et al*.^50^ studied the vibration shifts of BNC nanotube-based resonator in measuring an atomic-scale mass.

Due to the ultra-sensitivity of the resonator sensor to the mass to be measured, its experiments are sensitive to the environmental conditions, e.g., temperature and/or pressure, which reduce accuracy or even result in failure of the nanobalance. For a resonator-based balance, the measurement is available only when the nanobalance can capture the object to be measured. If the object moves at high speed, e.g., the nanoparticles are at high temperature, it may fail to capture the nanoparticle, and therefore, fails to measure the mass of the particle. In the present study, we focus on the capability of a BNC nanotube-based nanobeam in capturing a high-speed nanoparticle, i.e., we investigate the escape conditions of a nanoparticle, e.g., C_60_^51^, after collision with the BNC nanobeam.

## Model and Methodology

### Model

**Figure 1:**
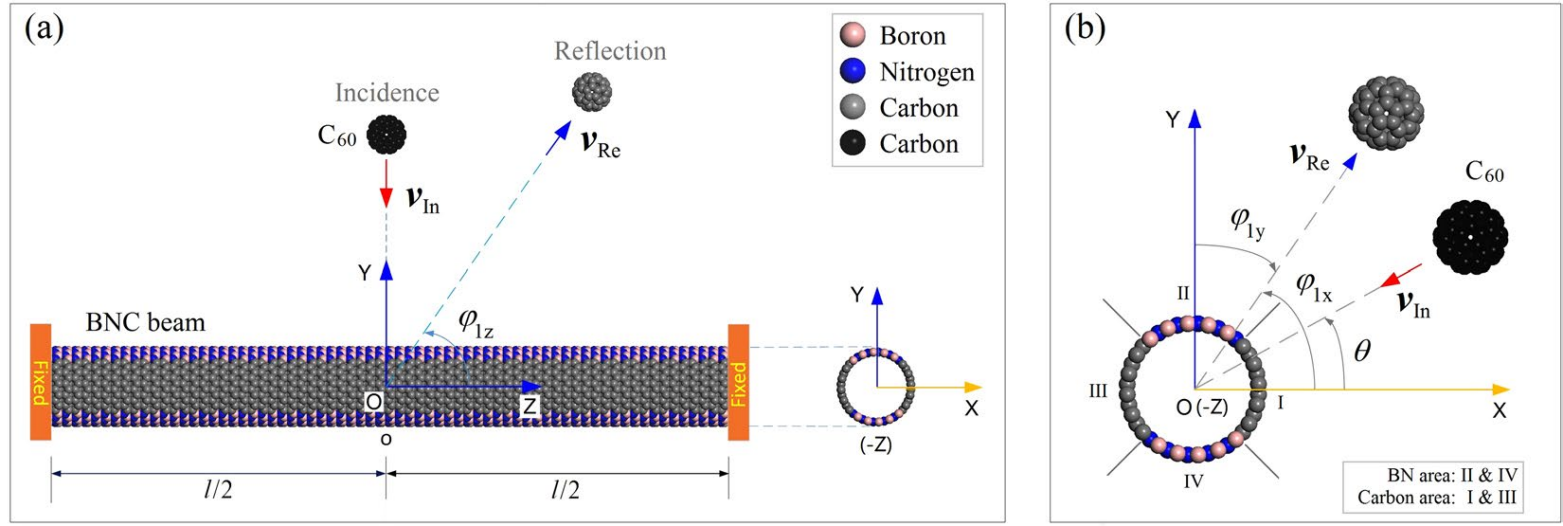
Geometric model of a clamped-clamped BNC nanotube beam under impact of C_60_. *l* = ~14.839 nm, which is the effective length of the (20, 0) nanotube beam with 50% of carbon atoms. *θ* is the angle of incidence of C_60_ with velocity of ***v***_In_. *φ*_1_ is the vector angle of reflection of C_60_ with velocity of ***v***_Re_. It contains three components, i.e., *φ*_1x_, *φ*_1y_ and *φ*_1z_. The vector of ***v***_In_ lies in x-y plane with arrow toward the centroid of beam. Planes X-Y, X-Z, and Y-Z are the symmetric planes of the carbon section of the beam. The initial distance between the mass center of C_60_ and beam is 3.0 nm. Before collision, C_60_ is in black, or in grey after impact. The cross section of the beam contains four areas with I&III as carbon area and II&IV as BN area.

Figure 1 gives the schematic of a BNC nanobeam under the impaction of a C_60_. The shell of the nanobeam contains four sections, i.e., two BN sections and two carbon sections. The path of incidence of C_60_ is in the x-y plane, which is the symmetric plane of the beam. The angle of incidence, *θ* varying from 0° to 90° with increment of 15° is investigated to show the effect of asymmetry of the cross-section of the nanobeam on the reflection of C_60_. Similarly, the incident velocity of C_60_, labeled as *v*_In_ and varying in a certain range is studied to find a critical value. Under the value, C_60_ will be attracted on the nanobeam. The critical value is called escape velocity of C_60_. When C_60_ escapes from nanobeam after impact, the velocity of reflection *v*_re_, may have three non-zero components along X-, Y-, and Z-axis. The components depend on the angle of reflection, i.e., *φ*_1_ in Fig. 1. For example, the component of *v*_Re_ along X-axis equal *v*_Re_ times cos(*φ*_1x_).

### Methodology

#### MD Simulation for collision between C_60_ and BNC nanobeam

To find the dynamic behavior of C_60_ during and after collision with the BNC nanobeam shown in Fig. 1, molecular dynamics simulations are carried out using the open source code Large Atomic/Molecular Massively Parallel Simulator (LAMMPS)^52,53^. In each simulation, the following steps are included,

1. Construct the nanobeam and C_60_, and layout the nanoball correspondingly, e.g., when *θ* = 15°, the initial position of the mass center of C_60_ is on the x-axis with coordinate of 30 Å;
2. Reshape the nanosystem by minimization of potential energy;
3. Fix both ends of the nanobeam and C_60_ during relaxation at canonical (NVT) ensemble. In this study, each relaxation keeps 20 ps and Nose-Hoover thermostat^54^ is used to adjust the velocity distribution;
4. After relaxation, change the system into a micro-canonical (NVE) ensemble, and release C_60_ and specify a constant velocity of incidence of the nanoball toward point O, i.e., the origin of the global coordinate system;
5. Calculate and record related dynamics features, including the coordinates and velocities of C_60_ and the mass center (point O*) of nanobeam;
6. Stop the movement of C_60_ after about 30–100 ps if it has escaped from the beam, and the nanobeam keeps running for further 0.3 ns;
7. Stop simulation.

In simulation, the interaction among atoms in the BNC nanobeam is estimated using Tersoff potential^55^; the interaction among the carbon atoms in the nanoball is evaluated using AIREBO potential^56^; and the interaction between the nanoball and nanobeam is described by Lennard-Jones (L-J) potential^57^. The timestep for integration is 0.001 ps.

#### Bisection algorithm for finding the critical escape velocity of C_60_ from nanobeam

**Figure 2:**
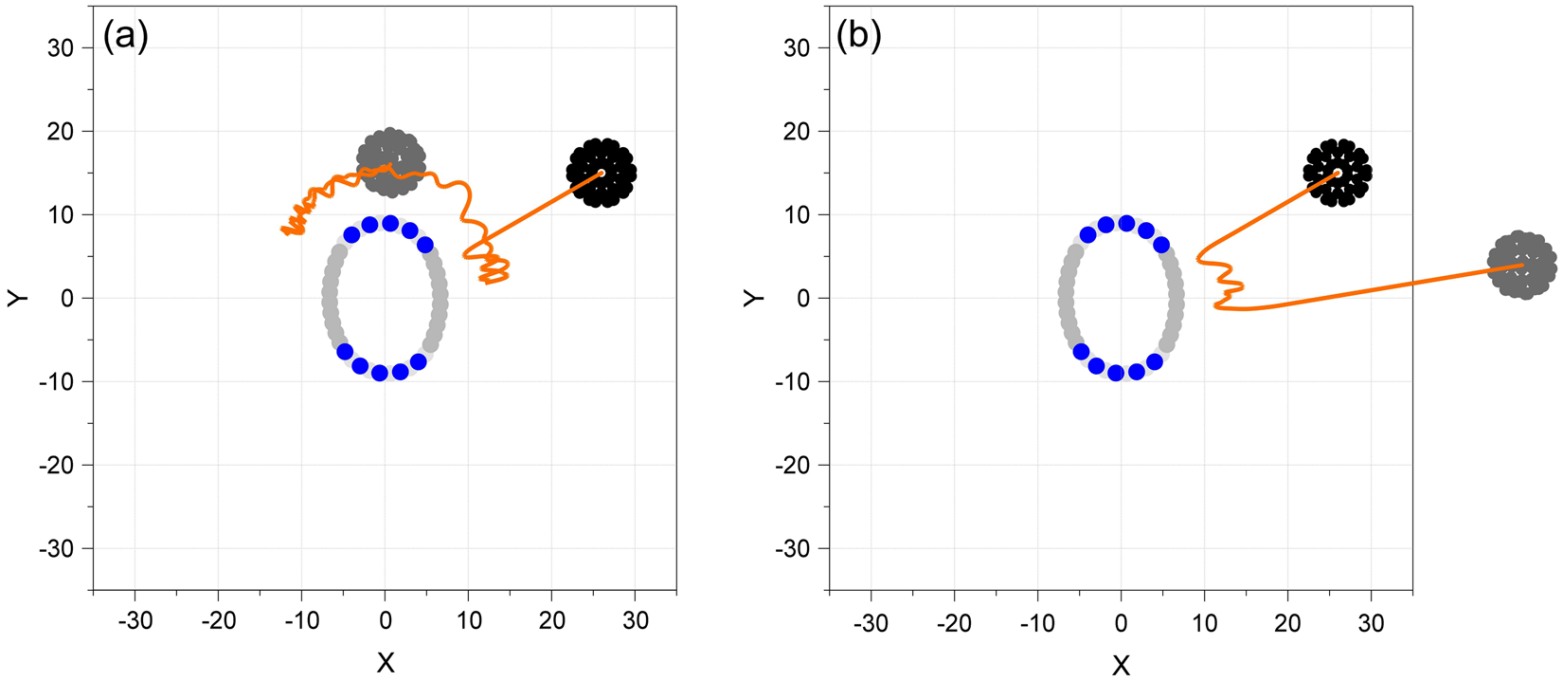
Trajectories of the fullerene nearby impact spot with an incident angle of *θ* = 30°. (**a**) *v*_In_ = 10.04 Å/ps during [0, 74.9]ps, (**b**) *v*_In_ = 12 Å/ps during [0, 50.6]ps (Movie 1). Unit of axis is Å. In beam, Boron atoms are in teal, Nitrogen atoms are in blue, and Carbon atoms are in light grey. C_60_ in black is before impact. After impact, C_60_ is in grey.

A fact to be demonstrated is that C_60_ may be attracted upon the surface of nanobeam when its velocity of incidence is too low. For example, in Fig. 2a, the incident nanoball (in black) with *θ* = 30° and *v*_In_ = 10.04 Å/ps is captured by the nanobeam. It can only move upon the surface of beam. If the velocity is high enough, e.g., *v*_In_ = 12 Å/ps, the nanoball can escape from the nanobeam (see Fig. 2b). Hence, we predict that there exists a value for incident velocity (*v*_In_), with which the nanoball can escape from the nanobeam after collision, but the velocity of reflection (*v*_Re_) is not higher than 1% of *v*_In_. To find this critical value of incident velocity, i.e., the minimal escape velocity (MEV), the bi-section algorithm is adopted, and five steps are built in the algorithm, i.e.,

(i) Determine the initial interval of *v*_Re_, e.g., [a, b] which satisfies Sign(a) × Sign(b) < 0;
(ii) Let c = (a + b)/2, calculate Sign(c);
(iii) If Sign(c) × Sign(a) > 0, let a = c; or let b = c;
(iv) Judge: if (b − a)/(a + b) < 0.1%, go to v); otherwise, go to ii);
(v) Stop and write down *v*_Re_^cr^ = c.

where Sign(*) = 1 if the distance between the mass center of C_60_ and the nanobeam, *d*_O*_, is higher than a critical value of distance within 0.3 ns, otherwise, Sign(*) = −1. The distance between the mass center of C_60_ and origin O is marked as *d*_O_. The radii of C_60_ and beam are 3.56 Å and 7.83 Å, respectively. The cutoff of L-J potential is 10 Å. By considering the deformation of the cross section of nanobeam, the critical value of distance is set to be 25 Å in the present study.

From the flowchart of MD simulation above, we know that the ensemble of the system shifts from NVT to NVE. The reason is that the nanobeam may be kept within a thermostat for a very long time for the relaxation of the system. Besides, it is known that if the nanoball can escape from the nanobeam surface after collision, the impact between the two components in the nanosystem needs no more than 100 ps (see Fig. 2b). During this period, the total energy of the system keeps unchanged. This is the reason for us to choose a microcanonical (NVE) ensemble for the system during collision.

#### Possible states of C_60_ during collision with nanobeam

**Figure 3:**
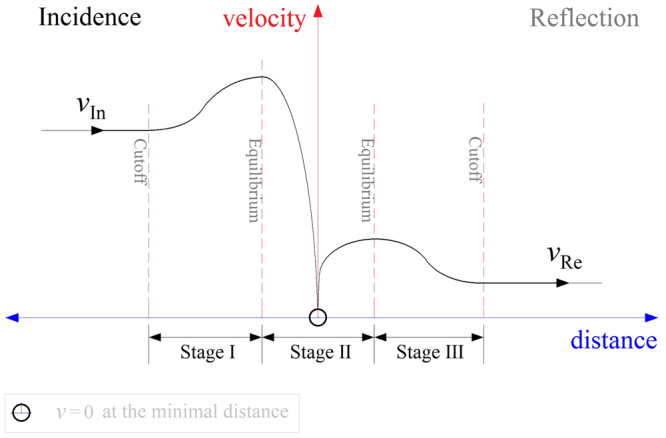
Schematic curve of velocity of C_60_ v.s. the surface distance between the nanoball and the nanobeam during collision.

In this study, we consider that the impact begins with the distance between the incident nanoball and the nanobeam being equal to the cutoff of L-J potential and ends with the same distance between the reflect nanoball and the nanobeam. The process of energy exchange between the nanoball and the nanobeam has three typical stages as shown in Fig. 3.

In stage I, the nanoball at a certain incident velocity is moving toward the nanobeam. In the period, the interaction between them is in the state of attraction when their distance is not less than the equilibrium distance, i.e., ~3.4 Å. In fact, the attraction continues even if the distance between them is slightly less than the equilibrium distance because of their space surfaces. During such attraction, the mass center of the nanoball is in accelerated motion, i.e., the velocity of C_60_ increases, but the variation of the velocity of C_60_ is not obvious because of the short acceleration duration. Meanwhile, the nanobeam is actuated to move due to the attraction. At this stage, the energy of the system changes from the surface (potential) energy to kinetic energy.

In stage II, the interaction between the nanoball and the nanobeam is in the state of repulsion. And before and after the relative stoppage of C_60_ on the surface of nanobeam, the velocity of C_60_ varies differently. For example, before stoppage, the nanoball is in the state of deceleration, i.e., the velocity is reduced. During the period, the kinetic energy together with part of surface energy is transformed into the deformation potential energy of the system. It is at the stoppage state, the surface distance between the nanoball and the nanobeam approaches minimum which is less than 3.4 Å. And during the stoppage, the nanoball incidence ends and the reflection starts. After stoppage, the repulsion accelerates the ball, and the nanoball obtains high velocity again. However, the stress wave on the nanobeam due to collision before stoppage starts propagating in the beam. Therefore, the reflect velocity of C_60_ is not as high as its incident velocity. But in this period, some potential energy turns back into the kinetic energy of C_60_ and the surface energy of the two components.

In stage III, the nanoball at a new velocity passes by the equilibrium position with respect to the surface of nanobeam. And the nanoball is attracted by the surface of nanobeam. Hence, the nanoball is back in a deceleration state. Before the reflect velocity of the C_60_ becomes zero, if the surface distance between the two components exceeds the 10 Å, we believe that the ball could escape from the attraction of nanobeam. Otherwise, the nanoball will be attracted back to the surface of nanoball again, i.e., the ball fails to escape.

#### Energy transformation during collision

To evaluate the transformation of the energy during collision, consider following equation,

1 $$E={{K}_{{\rm{m}}}}^{({\rm{b}}+{\rm{B}})}+{{K}_{{\rm{T}}}}^{({\rm{b}}+{\rm{B}})}+{{P}_{{\rm{d}}}}^{({\rm{b}}+{\rm{B}})}+{P}_{{\rm{s}}}={\rm{Constant}}$$

where *K*_m_^(b+B)^ is the kinetic energy of the mass centers of the nanoball (b) and nanobeam (B), *K*_T_ is the kinetic energy with respect to thermal vibration of atoms in the system, *P*_d_ is the deformation potential energy of the system, and *P*_s_ with the initial value of zero is the variation of the surface potential energy. *E* is not the total energy of the system due to the ignoring of the initial potential energy of the two components. During collision, the b components in *K*_T_ and *P*_d_ are very small and can be ignored. Therefore, Eq. (1) becomes

2 $$E\approx {{K}_{{\rm{m}}}}^{({\rm{b}}+{\rm{B}})}+{K}_{{\rm{T}}}^{{\rm{B}}}+{P}_{{\rm{d}}}^{{\rm{B}}}+{P}_{{\rm{s}}}={\rm{Constant}}\,1$$

with

3 $$\{\begin{array}{c}{{K}}_{{\rm{m}}}^{{\rm{b}}}\approx \frac{1}{2}{m}_{{\rm{bc}}}\times {v}_{{\rm{bc}}}^{2}\\ {{K}}_{{\rm{m}}}^{{\rm{B}}}\approx \frac{1}{2}{m}_{{\rm{Bc}}}\times {v}_{{\rm{Bc}}}^{2}\\ {K}_{{\rm{T}}}^{{\rm{B}}}=\frac{3}{2}{k}_{{\rm{B}}}\times N\times {T}_{{\rm{t}}},\end{array}$$

where *k*_B_ is the Boltzmann constant, *N* is the number of atoms on the nanobeam and *T*_t_ is the equivalent temperature of the system at t time. *m*_bc_ and *m*_Bc_ are the masses of the nanoball and the nanobeam, respectively. *v*_bc_ and *v*_Bc_ are the magnitudes of velocities of the mass centers of the nanoball and the nanobeam, respectively.

*P*_s_ is the difference between the potential energy of the current system and the total potential energy of isolated components. Commonly, it is negative. It reaches the minimum at the equilibrium state between the two components in the system. In the present model, the minimum depends on the location of the contact point during collision, e.g., −0.891 eV at *θ*  = 0° (carbon area I or III in Fig. 1) or −0.027 eV at *θ*  = 90° (BN area II or IV). If the contact point is nearby the C-BN interface, the value is between the two values. Hence, we predict that the nanoball can escape from the BN area easier than from the carbon area on the beam surface.

In the initial state of the system at NVE ensemble, the nanoball is out of the cutoff of the nanobeam. Hence, the total energy of the system is determined by the following equation,

4 $$E=\frac{1}{2}{m}_{{\rm{bc}}}\times {v}_{{\rm{In}}}^{2}+\frac{3}{2}{k}_{{\rm{B}}}\times N\times {T}_{0}.$$

Meanwhile, ***M***, the momentum of the system, keeps unchanged during collision, i.e.,

5 $${\boldsymbol{M}}={m}_{{\rm{bc}}}\cdot {{\bf{v}}}_{{\rm{In}}}={m}_{{\rm{bc}}}\cdot {{\bf{v}}}_{{\rm{bc}}}+{m}_{{\rm{Bc}}}\cdot {{\bf{v}}}_{{\rm{Bc}}}={\rm{Constant}}\,2$$

where ***v***_In_ = *v*_In_ × (cos*θ* sin*θ* 0). If the nanoball has a reflect velocity of ***v***_Re_ for escape, i.e., ***v***_Re_ = *v*_Re_ × (cos*φ*_1x_ cos*φ*_1y_ cos*φ*_1z_). From Eq. (5), the vector velocity of the nanobeam can be expressed as

6 $${{\bf{v}}}_{{\rm{Bc}}}=\frac{{m}_{{\rm{bc}}}}{{m}_{{\rm{Bc}}}}({{\bf{v}}}_{{\rm{In}}}-{{\bf{v}}}_{{\rm{Re}}})$$

Substituting Eqs (3, 4 and 6) in Eq. (2), we obtain the function of *v*_In_, i.e.,

7 $$\frac{1}{2}{m}_{{\rm{bc}}}\times {v}_{{\rm{Re}}}^{2}+\frac{{m}_{{\rm{bc}}}^{2}}{2{m}_{{\rm{Bc}}}}{({{\bf{v}}}_{{\rm{In}}}-{{\bf{v}}}_{\mathrm{Re}})}^{2}+\frac{3}{2}{k}_{{\rm{B}}}\times N\times {T}_{{\rm{t}}}+{P}_{{\rm{d}}}^{{\rm{B}}}+{P}_{{\rm{s}}}\approx \frac{1}{2}{m}_{{\rm{bc}}}\times {v}_{{\rm{In}}}^{2}+\frac{3}{2}{k}_{{\rm{B}}}\times N\times {T}_{0}$$

or simplified as

8 $$\frac{{m}_{{\rm{bc}}}}{2{m}_{{\rm{Bc}}}}[({m}_{{\rm{Bc}}}-{m}_{{\rm{bc}}}){v}_{{\rm{In}}}^{2}-({m}_{{\rm{Bc}}}+{m}_{{\rm{bc}}}){v}_{{\rm{Re}}}^{2}-2{m}_{{\rm{bc}}}{{\bf{v}}}_{{\rm{In}}}\cdot {{\bf{v}}}_{\mathrm{Re}}]\approx \frac{3}{2}{k}_{{\rm{B}}}\times N\times ({T}_{{\rm{t}}}-{T}_{0})+{P}_{{\rm{d}}}^{{\rm{B}}}+{P}_{{\rm{s}}}$$

When the incident velocity of nanoball, i.e., *v*_In_, reaches its critical value of escape velocity, the value of *v*_Re_ can be considered as zero, the above equation yields

9 $$\frac{{m}_{{\rm{bc}}}({m}_{{\rm{Bc}}}-{m}_{{\rm{bc}}})}{2{m}_{{\rm{Bc}}}}{v}_{{\rm{In}}}^{2}\approx \frac{3}{2}{k}_{{\rm{B}}}\times N\times ({T}_{{\rm{t}}}-{T}_{0})+{P}_{{\rm{d}}}^{{\rm{B}}}+{P}_{{\rm{s}}}.$$

The first and the second items of the left part of Eq. (9) are determined by two factors. One is the length of nanobeam and its boundary conditions, and the other is the local bending rigidity of the shell of nanobeam. For example, for a stiffer shell of a longer nanobeam, the first item of the left part will be higher due to wave propagation in the shell. For a softer shell with higher length, the second item, i.e., *P*_d_^B^, will be higher. The two parts should be no less than zero during collision. The third item of the left part, which only depends on the location of the contact point during collision, is negative. If the mass of nanoball is far less than that of nanobeam, the following relationship can be obtained, i.e.,

10 $$\frac{1}{2}{m}_{{\rm{bc}}}\times {v}_{{\rm{In}}}^{2}\approx \frac{3}{2}{k}_{{\rm{B}}}\times N\times ({T}_{{\rm{t}}}-{T}_{0})+{P}_{{\rm{d}}}^{{\rm{B}}}+{P}_{{\rm{s}}},$$

Eq. (10) can also be transformed into the following form,

11 $$\frac{1}{2}{m}_{{\rm{bc}}}\times {v}_{{\rm{In}}}^{2}-{P}_{{\rm{s}}}\approx \frac{3}{2}{k}_{{\rm{B}}}\times N\times ({T}_{{\rm{t}}}-{T}_{0})+{P}_{{\rm{d}}}^{{\rm{B}}},$$

The first item of the left part is obtained when the incident velocity and the mass of the impact object is specified. The second item can be estimated when the beam surface and the angle of incidence are chosen. Hence, the left part of Eq. (11) can be considered as the input energy of the system. The right part of Eq. (11) contains both the temperature-related kinetic energy and the deformation potential energy of the nanobeam. Obviously, *T*_t_ should not be less than *T*_0_ because the vibration of the atoms in the nanobeam becomes more drastic under after collision. And the value of *P*_d_^B^ approaches the maximum when *T*_t_ = *T*_0_, which indicates that the total input energy has been transformed into deformation potential energy of the beam.

Clearly, the above analysis is based on the collision between two deformable bodies, and the local thermal vibration of atoms on both components of the system is not considered. As we know, the vibration of atoms is more drastic at higher temperature. During collision, the repulsion between both components mainly happens among the surface atoms nearby the contact point. Owing to the drastic vibration of the surface atoms on the nanobeam, the nanoball can obtain a higher velocity of reflection as comparing to the surface without thermal vibration. Hence, we predict that at higher temperature, the critical value of escape velocity of C_60_ will be lower.

**Figure 4:**
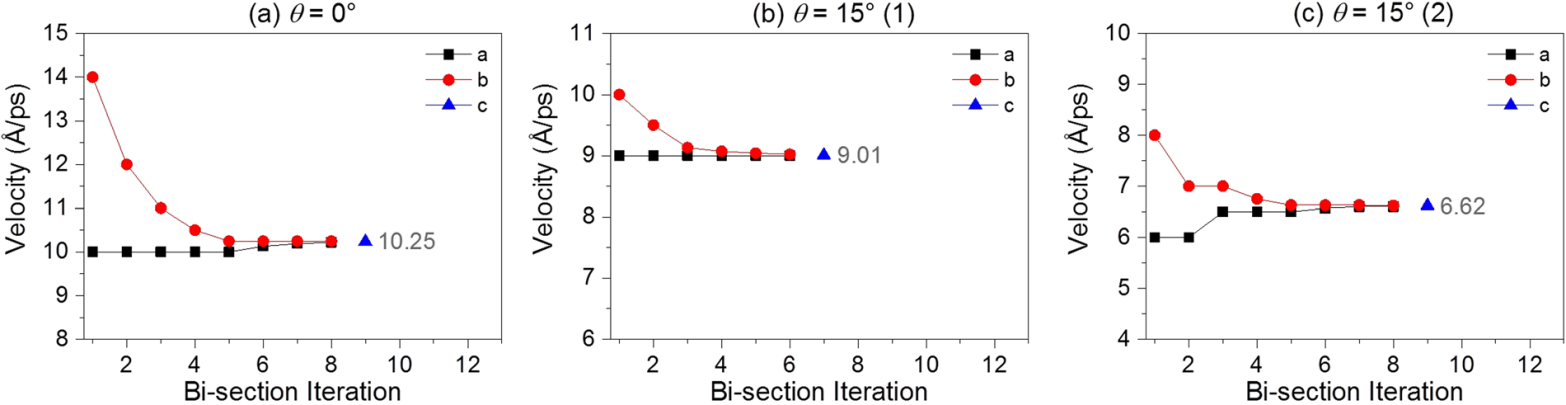
Schematic of local vibration modes of the beam surface with respect to the same C_60_.

One more factor should also be demonstrated. The factor is the deformation-induced vibration of the surface of nanobeam. For example, the nanoball after stoppage at stage II, the deformation-induced vibration of the surface might lead to the reduction of the surface distance between the two components. In this situation, the nanoball will obtain higher acceleration, which means that the reflect velocity of C_60_ becomes higher. On the contrary, if the deformation-induced vibration leads to larger distance between both components, the reflect velocity of C_60_ becomes lower. The nanoball might be captured by the nanobeam at the third stage. However, the local vibration of the surface of nanobeam at the contact point depends on the bending rigidity of the beam along the collision direction. For example, if the contact point on the nanobeam is located in the carbon area, i.e., encircled by carbon atoms, the vibration frequency is higher but the amplitude could be lower as comparing to that in the BN area. Clearly, the amplitude of vibration influences the acceleration of C_60_ significantly. For example, if the vibration direction of the surface of nanobeam is aligned with the reflection direction, the attraction of nanobeam can keep for a longer time. The ball could be captured by the nanobeam. For the nanoball with the same reflect velocity (Fig. 4a), it could escape from the nanobeam whose surface at the contact point vibrates along the opposite direction (Fig. 4b). This implies that the critical value of C_60_ may not be unique. Correspondingly, the escape time depends on the geometry and boundary conditions of the nanobeam in which the phonon propagation and reflection happen during collision.

#### Multivariate analysis of variance

From the discussion above on energy transformation and the stages of C_60_ during collision, local thermal vibration of atoms on the beam surface may be of significance to the escape of the nanoball. The local vibration of atoms mainly depends on three factors. The first factor is temperature, which influences the initial distribution of velocities of atoms at the beam surface. The second one is the geometry of nanobeam together with boundary conditions, which affect the wave propagation during collision. And the final is the way of incidence of the nanoball. Hence, multivariate analysis of variance (ANOVA) is necessary to find the significance of the factors on the dynamic behavior of C_60_. In the present study, we focus on the temperature-related random numbers and incidence of C_60_ on the reflection angle (*φ*) of the nanoball.

In a factor analysis at *k* levels, and each level containing *r* observations, we define *X*_*ij*_ as the *j*^th^ observation at the *i*^th^ level. The significant difference of a factor, labeled as “A”, with respect to the observations can be calculated using the following formulation,

12 $${F}_{{\rm{A}}}=\frac{{S}_{{\rm{A}}}^{2}}{{S}_{e}^{2}}=(\frac{S{S}_{{\rm{A}}}}{k-1})/(\frac{S{S}_{e}}{k(r-1)}),$$

where *SS*_A_ and *SS*_e_ are the mean square sums (SS) of the factor A and error, respectively, and can be calculated using following equations:

13 $$S{S}_{A}=\frac{1}{r}\sum _{j=1}^{r}{T}_{j}^{2}-\frac{{T}^{2}}{k\cdot r};\quad S{S}_{e}=\sum _{i=1}^{k}\sum _{j=1}^{r}{X}_{ij}^{2}-\frac{1}{r}\sum _{i=1}^{k}{T}_{i}^{2},$$

and

14 $$T=\sum _{i=1}^{k}{T}_{i}=\sum _{i=1}^{k}\sum _{j=1}^{r}{X}_{ij}.$$

when *F* > *F*_*P≤*0.05_(*k* − 1, *k*(*r* − 1)), the factor A is significant to the observations. The commercial software Statistical Analysis System (SAS)^58,59^ is used for the factor analysis.

## Numerical Experiments and Discussion

From Fig. 2, we find that the central cross section of the BNC beam has become an ellipse, rather than an ideal circular (Fig. 1b). Hence, the path of C_60_ after collision to the nanobeam will not be aligned with that before collision even if the surface of beam is a smooth shell. On the other hand, the nanoball may be attracted by and moves on the surface of beam if the velocity of incidence of nanoball is lower than a certain value (Fig. 2a). Therefore, the trajectory of C_60_ with initially specified velocity (*v*_In_) depends on the interaction between the nanoball and beam. Except the attraction of beam, the vibration of beam surface also has influence on the path of nanoball. And the vibration of beam surface contains two aspects, one is caused by the collision between the nanobeam and the nanoball, and the other is the thermal vibration of the atoms on beam surface. Due to these reasons, we will focus on the critical value of incident velocity, i.e., MEV, of C_60_ together with such factors as angle of incidence and temperature.

### Determination of the MEV of C_60_ by bi-section algorithm

**Figure 5:**
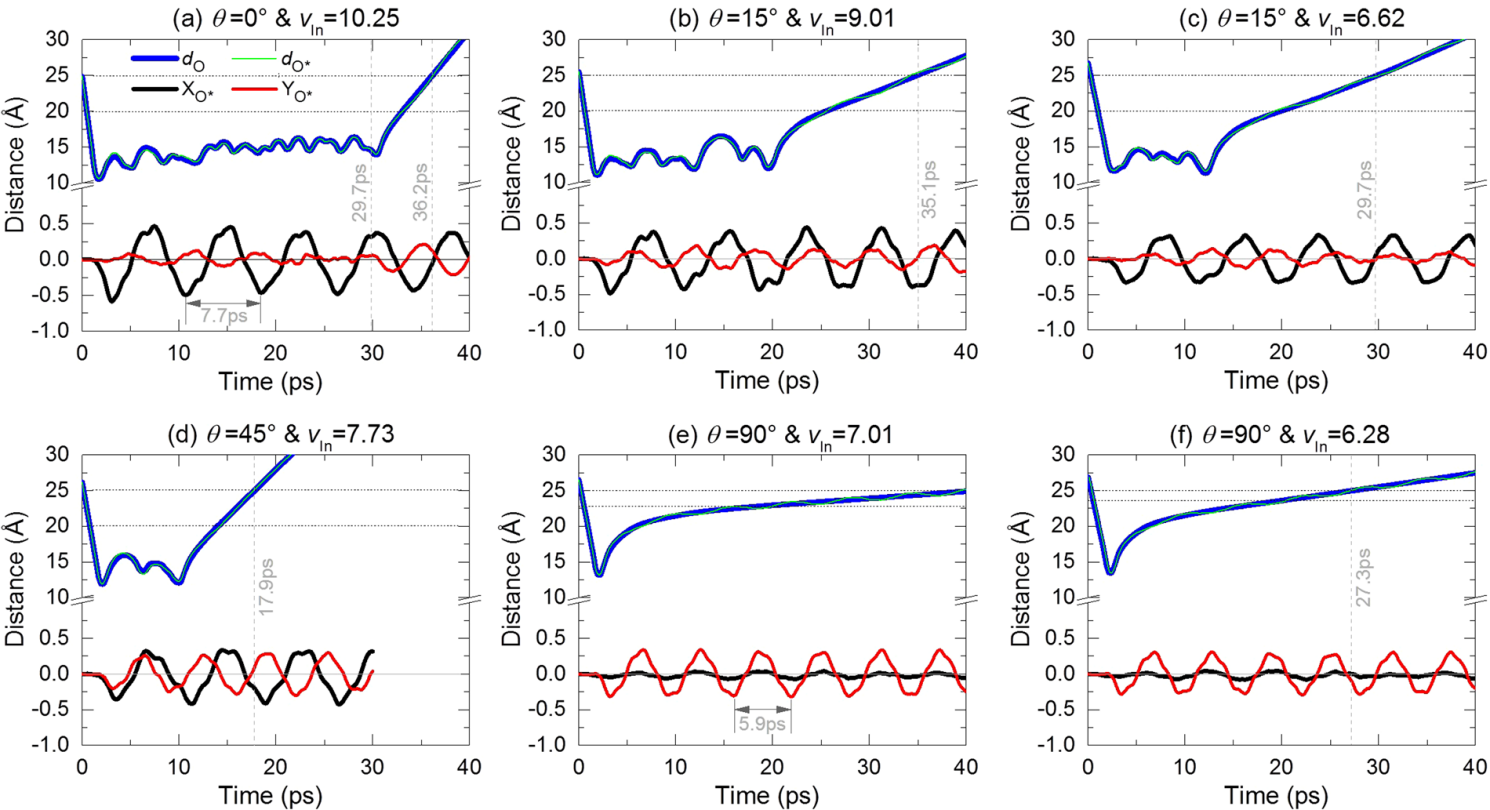
The iteration process for searching for the escape velocity of C60 which impacts the BNC nanobeam. (**a**) *θ* = 0°, (**b**) and (**c**) *θ* = 15°. (More data are given as supporting material in).

Using bi-section algorithm, we obtain the MEVs of C_60_, which impacts the BNC nanobeam from different directions at 8 K. And the results are listed in Table 1. The process for finding the values of MEVs by bi-section method can be seen in Fig. 5, and related supporting material. It is found that the MEV may not be isolated even if the angle of incidence of C_60_ is the same. For example, when *θ* = 15°, the nanoball with 10.25 Å/ps or with 6.62 Å/ps, C_60_ can escape from the nanobeam. However, if the nanoball has a slightly lower velocity than the MEV, the nanoball will be captured by the nanobeam due to surface attraction. To reveal the mechanism of this phenomenon, we examine the histories of the relative positions and the variation of configurations of both components in the system with different incidence of C_60_ at different speed. The interaction between the nanoball and the nanobeam is also traced during their collision.

**Table 1:** The escape velocities of C_60_ impacting the BNC nanobeam at 8 K. Unit: Å/ps.

*θ*	0°	15°	30°	45°	60°	75°	90°
*v*_In(1)_	10.25	9.01	10.07	7.73	10.29	8.92	7.01
*v*_In(2)_	×	6.62	7.94	×	7.23	6.31	6.28

**Figure 6:**
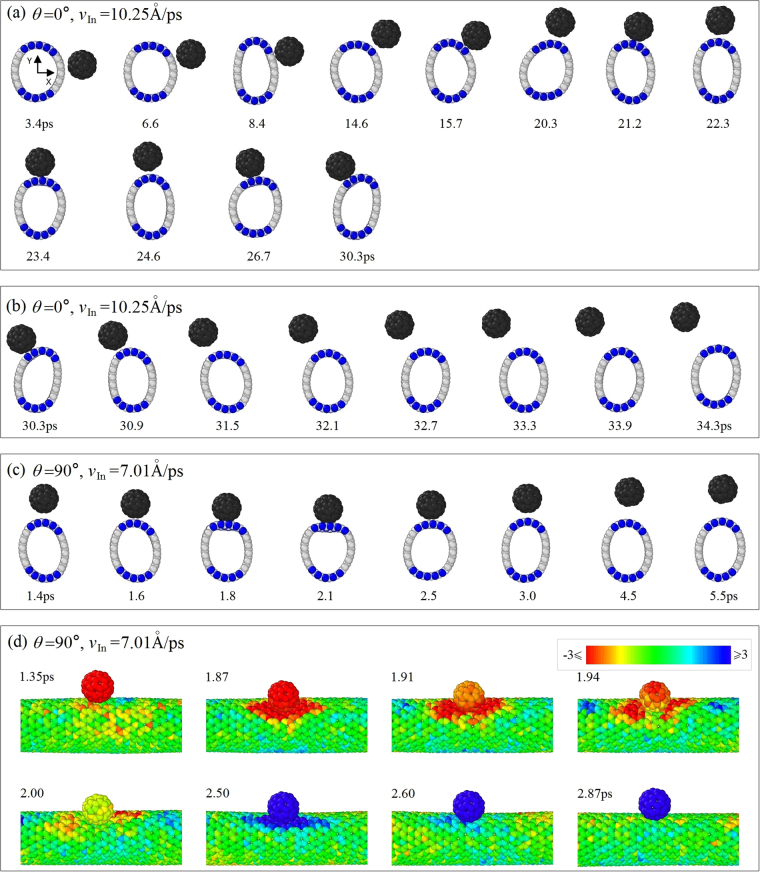
Distance histories after releasing C_60_ to impact the BNC nanobeam from different direction. Label “*d*_O_” means the distance between the mass center of C_60_ and the initial origin (point O). “*d*_O*_” is the mass center distance between C_60_ and beam. “X_O*_” and “Y_O*_” are the x- and y-coordinate of mass center of nanobeam, respectively. (More data are given as supporting material in S-Figure 1 and S-Figure 2).

Figure 6 shows the histories of *d*_O_, *d*_O*_, X_O*_ and Y_O*_ within the first 40 ps of the system. *d*_O_ (blue line) and *d*_O*_ (green line) are the distance from the mass center of C_60_ to the initial and current mass center of the BNC nanobeam. The two curves have slight difference which means the relative displacement of their mass centers. The difference is mainly caused by the deformation of the beam surface at contact point during collision, rather than by the vibration of the mass center of nanobeam which is illustrated by the curves of X_O*_ and Y_O*_. From Fig. 6 we can also find that the nanoball needs longer time to escape from the nanobeam (*d*_O*_ > 25 Å) when the angle of incidence is lower, i.e., the angle between the path of incidence and the X-axis. Besides, in general, the MEV of C_60_ decreases with the increasing of the angle of incidence. From the characteristics of the blue curve, we know that the curve becomes a straight line much earlier before *d*_O*_ > 25 Å happening. A straight line of *d*_O_ means that the reflect velocity of C_60_ is a constant. Hence, the straight part of the blue curve, e.g., between 29.7 and 36.2 ps in Fig. 6a, demonstrates that the ball has escaped from the attraction of the nanobeam. When the angle of incidence is small, the straight part starts from ~20 Å. However, the straight part begins from ~23 Å if the angle of incidence is 90°. The difference is mainly caused by the ellipse cross section of the nanobeam after full relaxation (Fig. 2).

From the curves of X_O*_ and Y_O*_ in Fig. 6, the vibration period of the nanobeam along different direction is also not identical. For example, in Fig. 6a, the vibration period of the beam is ~7.7 ps when *θ* = 0°, while, ~5.9 ps when *θ* = 90° in Fig. 6e. The difference is caused by different bending rigidities of the cross section of beam (Fig. 2) along different direction. Clearly, vibration period of the beam in the YOZ plane being lower than that in the XOZ plane implies that the bending rigidity of the cross section in the YOZ plane (when collided by C_60_ with *θ* = 90°) is higher than that in the XOZ plane.

**Figure 7:**
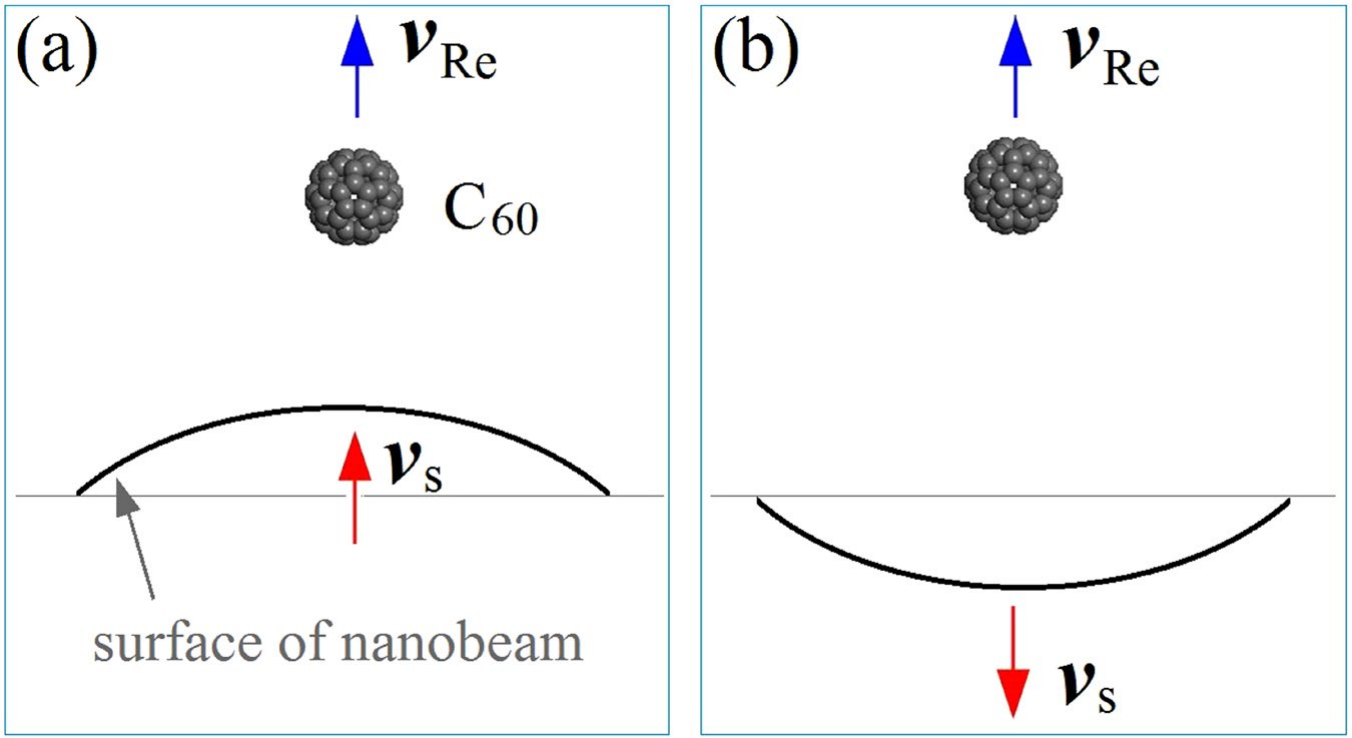
The snapshots of the system in the XOY plane during collision. (**a**) Sliding of C_60_ on beam surface, (**b**) escape from beam surface when *θ* = 0°, *v*_In_ = 10.25 Å/ps. (**c**) Escape of C_60_ from beam surface when, (**d**) local velocity distribution of atoms during collision when *θ* = 90° and *v*_In_ = 7.01 Å/ps (Movie 2). Red means velocity is no higher than −3Å/ps, blue means no less than 3 Å/ps.

Another important phenomenon, i.e., the curve of *d*_O_ waving between 10 to 20 Å when the angle of incidence is low, needs to be demonstrated. To show the mechanism, we list the snapshots of the cross section of the beam and the nanoball during their collision with angle of incidence of 0° and incident velocity of 10.25 Å/ps as shown in Fig. 7. Figure 7a shows that the nanoball slides on the beam surface from the right side (carbon area I) at 3.4 ps to the top BN area II after 14.6 ps. It can also be found that the nanoball jumps on the beam surface during sliding. For instance, the gap between their surfaces fluctuates continuously. In fact, a snapshot with low surface gap is captured at a trough of the curve of *d*_O_, whilst, a snapshot with high surface gap is obtained at a crest. During sliding, the shape of cross section of the nanobeam varies greatly due to the weak bending rigidity of the BN sections. When the nanoball moves to the interface between the top BN area II and the left carbon area III, it does not move further (Fig. 7b). Nearby the interface, the nanoball jumps together with the beam surface. At the time of 32.1 ps, the nanoball is pushed away from the beam surface and starts escape. It is mainly because the beam surface at the BN area waves with higher amplitude, however, it provides weaker attraction to C_60_ due to sharp curvature.

Moreover, during waving of beam surface, the atoms in the surface also has thermal vibration, which may provide the nanoball a sharp jump of velocity. Hence, the nanoball has chance to escape from the beam surface. For instance, when C60 moves towards the beam surface from top (*θ* = 90°, *v*_In_ = 7.01 Å/ps) (Fig. 7c), the velocity distribution of atoms on the beam surface at 1.35 ps (Fig. 7d) is different from that at 1.87 ps. The red area on the beam surface means that the atoms move with the C60 along the same direction. From 1.91 to 2.00 ps, the velocity of the ball drops to be zero. At 2.5 ps, both the nanoball and the contact point on the beam surface shows in blue color, which means that the nanoball being bounced back by the vibration of the beam surface. Later, e.g., at 2.6 ps or 2.97 ps, the ball keeps moving away from the beam surface. Hence, the local velocity at the contact point on the beam surface determines the escape state of the nanoball.

**Figure 8:**
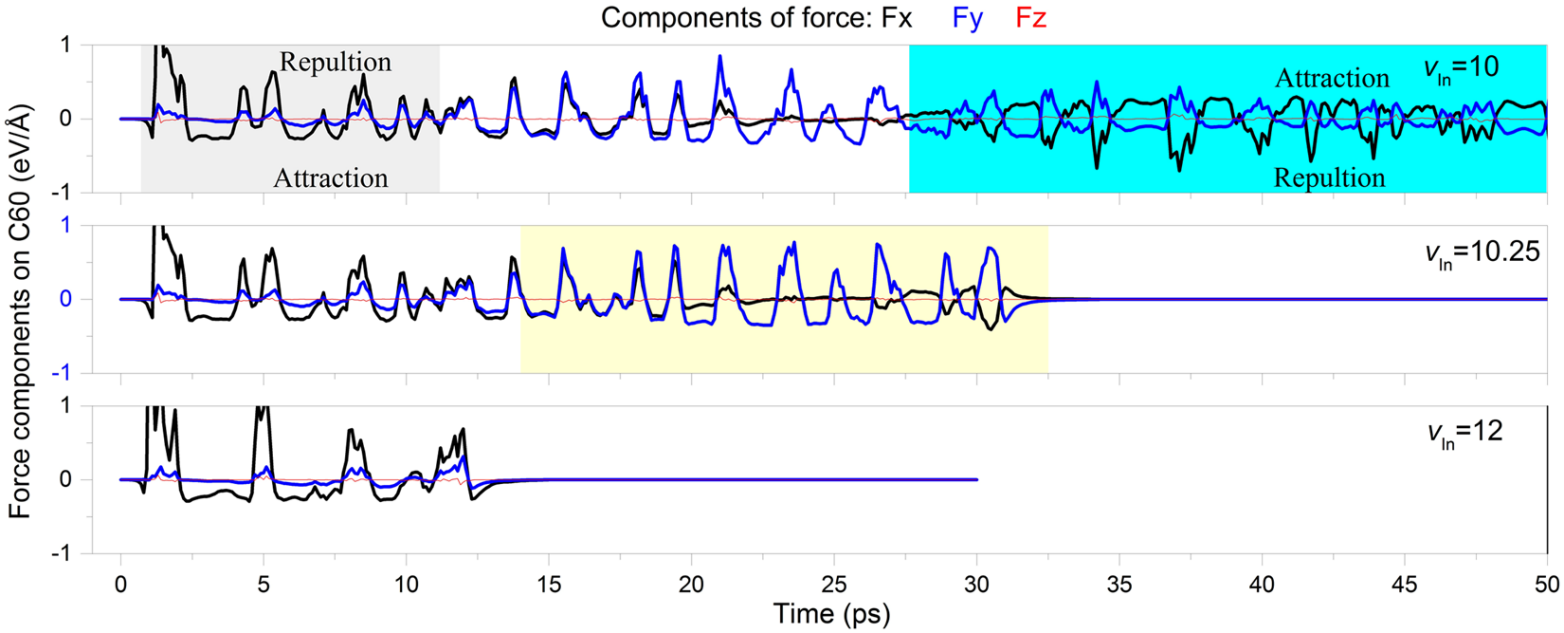
Histories of components of force on the mass center of C_60_ with *θ* = 0°. Fx, Fy and Fz are the x-, y- and z-component of force of the nanobeam acting upon the naonball, respectively. Sharp vibration of curve indicates repulsion and smooth vibration of curve means attraction.

From above analysis, it is clear that the escape of C_60_ depends on both angle of incidence and incident velocity. Especially, when the value of the incident velocity is at or near the MEV, the nanoball may slides on the surface of beam, rather than escape directly. During the period, the interaction between the nanoball and the nanobeam determines the escape condition of C_60_. To show the interaction, we give the curves of force on the mass center of C_60_ with *θ* = 0° in Fig. 8. When the incident velocity of C_60_ is lower than the MEV, say, 10 Å/ps, the x-component of force (Fx/black curve) fluctuates drastically during the first ~10 ps. During the same period, both y-component (Fy/blue curve) and z-component (Fz/red curve) wave slightly. It indicates that the nanoball is accelerated mainly along x-axis. As positive value means repulsion and negative means attraction when the mass center of C_60_ locates in the first quadrant (snapshots at 3.4/6.6/8.4 ps in Fig. 2), the amplitude of fluctuation of the repulsion force is much higher than that of attraction. During 12 and 24 ps, the fluctuation of F_y_ is much higher than that of F_x_. However, due to the beam surface has lower amplitude of vibration during collision, C_60_ cannot escape from the beam surface.

If the incident velocity is no less than MEV, the nanoball can escape from the beam surface according to the straight lines of F_x_ and F_y_. For example, when *v*_In_ = MEV = 10.25 Å/ps, both F_x_ and F_y_ tend to be zero after ~33 ps (see the mid-layer of Fig. 8). One can also find that F_y_ (blue curve) fluctuates more obvious than F_x_. According to the snapshots in Fig. 7b, the nanoball escapes from the surface at the upper BN section due to strong repulsion. When *v*_In_ = 12 Å/ps > MEV (see the bottom-layer of Fig. 8), the nanoball escapes from the beam surface much earlier as comparing with the nanoball having MEV. Meanwhile, before escape of nanoball, both F_x_ and F_y_ has the same characteristic of variation, i.e., simultaneous repulsion or attraction, which is different from that when *v*_In_ = MEV. But the peak amplitudes of force components are not obviously higher when *v*_In_ is higher. The reason is that the repulsive force depends on the surface distance between the nanoball and the nanobeam, and the surface distance is determined by the thermal vibration of atoms on the beam surface.

In any case of Fig. 8, the variation of F_z_ is far less than that of F_x_ or F_y_. It implies that the discretization of the beam surface has slight influence on the impacting nanoball during collision. Hence, *φ*_1z_, the x-component of the reflection angle of C_60_ may have small difference from 90°.

### Effect of the initial velocity distribution of beam surface on the reflection angle of C_60_

From the analysis above, it can be seen that the local mode and velocity of beam surface (Fig. 4) have obvious influence on the escape of C_60_. To show the influence we calculate the components of reflection angle, i.e., *φ*_1x_, *φ*_1y_ and *φ*_1z_, of C_60_ with *v*_In_ > MEV after collision with the nanobeam having different initial velocity distributions. In the present study, Nose-Hoover thermostat is used to specify the initial velocities of atoms by obeying Gaussian distribution. The initial velocity distributions of beam surface are obtained by specifying different random numbers.

Together with random numbers, the angle of incidence is involved in measuring the effect of velocity distribution on the reflection angle of C_60_. In this case, six random numbers (factor A), four angle of incidences (factor B) with *v*_In_ = 25 Å/ps are involved in experiments. The nanoball can escape from the beam surface and the components of reflection angles are listed in Table 2.

**Table 2:** Reflection angles of C60 at different conditions with T = 8 K. Random numbers (RN): A1 = 1976325; A2 = 6491263; A3 = 9061845; A4 = 324189; A5 = 2691534; A6 = 4928459. B1 = 25 Å/ps; B2 = 27 Å/ps; B3 = 30 Å/ps. Units: reflection angle *φ*/(°), velocity/(Å/ps).

RN	*v*_In_	C1 *θ* = 0°			C2 *θ* = 30°			C3 *θ* = 60°			C4 *θ* = 90°		
		*φ*_1x_	*φ*_1y_	*φ*_1z_	*φ*_1x_	*φ*_1y_	*φ*_1z_	*φ*_1x_	*φ*_1y_	*φ*_1z_	*φ*_1x_	*φ*_1y_	*φ*_1z_
A1	B1	**−12**.**46**	102.46	90.38	−39.43	129.34	92.19	−**137**.**75**	228.29	95.57	112.81	−23.15	93.79
	B2	−11.75	101.74	90.36	−39.73	129.63	92.47	−**124**.**38**	215.00	95.76	113.83	−22.99	93.88
	B3	−10.49	100.49	90.35	−41.51	131.37	92.88	−63.69	153.08	95.33	107.28	−17.95	**94**.**71**
A2	B1	7.22	82.82	89.19	−18.95	108.89	88.55	−6.93	95.54°	85.85°	85.72°	5.60°	93.61
	B2	−1.20	91.14	90.40	−23.99	113.98	89.24	−8.48	96.82	84.98	91.70	2.81	92.24
	B3	−3.40	93.40	89.98	−31.12	121.12	90.12	−11.10	98.70	84.00	100.74	−5.11	89.94
A3	B1	**10**.**14**	79.86	90.13	−13.75	103.68	91.36	−12.60	102.41	87.85	55.75	34.35	92.36
	B2	9.58	80.42	90.34	−15.52	105.51	90.70	−13.27	103.05	87.60	55.55	34.59	92.80
	B3	8.97	81.04	90.42	−19.52	109.52	89.77	−15.44	105.14	87.00	55.80	34.38	93.12
A4	B1	4.35	85.66	89.63	−21.30	111.19	92.05	−19.55	109.45	91.93	82.98	7.41	92.33
	B2	3.28	86.73	89.73	−23.81	113.70	92.16	−18.34	107.01	92.24	83.64	6.94	92.77
	B3	−1.94	91.73	90.87	−29.53	119.33	93.13	−16.38	106.17	92.53	84.10	6.54	92.80
A5	B1	−3.61	93.21	91.64	−31.05	121.04	89.38	−30.68	119.91	83.76	83.33	6.78	91.18
	B2	2.90	87.10	90.03	−30.54	120.51	88.81	−32.38	121.51	83.29	79.95	10.16	91.48
	B3	4.08	85.93	90.28	−28.48	118.44	88.54	−28.91	118.15	83.96	75.95	14.14	91.50
A6	B1	−8.66	98.66	89.95	−36.09	126.05	88.71	−**145**.**34**	238.39	77.25	114.22	−24.22	89.75
	B2	−7.80	97.80	89.68	−37.47	127.45	88.99	42.61	49.36	79.37	114.13	−24.19	88.38
	B3	−6.91	96.88	89.33	−39.91	129.90	89.18	−**96**.**10**	197.48	73.68	114.88	−25.06	**87**.**22**

From Table 2 one can find that the x-component of the reflection angle of C_60_ is between −12.46° and 10.14° when *θ* = 0°. Such obvious divergence of the reflection angle indicates that the initial velocity distribution of the atoms in the nanobeam does influence the escape of C_60_. The phenomenon, actually, demonstrates the low accuracy of the reflection angle of C_60_ in a real collision experiment, i.e., one cannot measure the path of incidence of a light nanoparticle, e.g., C_60_. By checking the values of *φ*_1x_ with respect to different values of *θ*, the divergence of *φ*_1x_ can also be found. Especially, when *θ* = 60° or 90°, the divergence of *φ*_1x_ is even higher. Oppositely, the values of *φ*_1z_ are in the interval of [87.22°, 94.71°], i.e., very close to 90°, on condition that *θ* is not 60°. It means that the reflection path has a slight deviation from the XOY plane. The deviation also approves our previous theoretical prediction.

To show the significances of the factors, i.e., RN and *v*_In_, on the reflection angle, the multivariate ANOVA is carried out. The results of ANOVA are shown in Table 3. For the case of *θ* = 0°, the determination coefficient (*R*^2^) is 0.87762. And the *P* value is 0.0007 < 0.01. Hence, the results are acceptable. Particularly, the *P* value with respect to RN is only 0.0003, which indicates that RN is the major factor which influences the value of *φ*_1x_. Similar conclusion can be obtained when *θ* = 90°. If *θ* = 30°, *R*^2^ > 0.95 and *P* values are less than 0.05. Hence, both factors influence the value of *φ*_1x_. But the influence of RN is more significant.

**Table 3:** ANOVA of *φ*_1x_. MV is the mean value of *φ*_1x_. DOF is the degree of freedom.

*θ*	Source	DOF	SS	Mean square	*F* value	*P* value (*P*_r_ > *F*)	MV
0°	Model	7	861.16	123.02	10.24	0.0007	−0.98
	RN	5	857.55	171.45	14.28	0.0003	
	*v*_In_	2	3.91	1.96	0.16	0.8518	
	Error	10	120.09	12.01			
	Total	17	981.25		*R*^2^ = 0.87762		
30°	Model	7	1282.52	183.22	27.34	<0.0001	−28.98
	RN	5	1207.99	241.60	36.05	<0.0001	
	*v*_In_	2	74.54	37.27	5.56	0.0238	
	Error	10	67.02	6.70			
	Total	17	1349.54		*R*^2^ = 0.95034		
60°	Model	7	26192.68	3741.81	1.99	0.1561	−41.04
	RN	5	22852.10	4570.42	2.43	0.1086	
	*v*_In_	2	3340.57	1670.29	0.89	0.4436	
	Error	10	18796.25	1879.62			
	Total	17	44988.92		*R*^2^ = 0.58203		
90°	Model	7	7139.14	1019.88	61.53	<0.0001	89.58
	RN	5	7137.39	1427.48	86.12	<0.0001	
	*v*_In_	2	1.75	0.87	0.05	0.9489	
	Error	10	165.76	16.58			
	Total	17	7304.90		*R*^2^ = 0.97731		

The ANOVA results with respect to *θ* = 60° shown in Table 3 indicate following two facts. One is that *R*^2^ is less than 0.6. The other is that *P* value with respect to either RN or *v*_In_, is higher than 0.05. It means that the ANOVA results are conditionally acceptable. Actually, from the results shown in Table 2, we know that the values of *φ*_1x_ with respect to *θ* = 60° are greater than any other cases. The two factors must have great influence on the reflection angle of C_60_ after collision. When we observe the whole process of collision with *θ* = 60° (C3) at the conditions of (A1 < B1, B2 > Movie 3, and A6 < B1 > Movie 4), C60 firstly slides on the beam surface and later escapes at the third quadrant of XOY. Hence, the statistical results are poor.

### Effect of temperature on escape of C_60_

#### The MEV of C_60_ at colliding with the nanobeam with different temperature

As temperature is significant to the escape of C60, we search for the MEV of C60 at different temperature and list the results in Table 4. It indicates that the value of MEV decreases rapidly with the increasing of temperature. For example, at ambient temperature, the value of MEV is only about 20~25% of that at 8 K. If temperature reaches 500 K, the value of MEV is only 0.39 Å/ps, i.e., 39 m/s when *θ* = 0°. Hence, the attraction of beam surface reduces drastically when temperature is high. It also verifies our previous prediction, i.e., the nanoball can escape easier from the beam surface at higher temperature.

**Table 4:** The MEVs of C_60_ as colliding with the nanobeam at different temperature with the same RN. Unit: velocity/(Å/ps)

*θ*		T = 8 K	T = 100 K	T = 300 K	T = 500 K
0°	*v*_In(1)_	10.25	6.62	2.2	0.39
	*v*_In(2)_		×	2.13	×
90°	*v*_In(1)_	7.01	8.51	2.08	0.7
	*v*_In(2)_	6.28	4.24	×	×

#### Effect of temperature on the reflection angle C_60_

To show the significance of both temperature and RN on the reflection angle of C_60_, we set *v*_In_ = 20 Å/ps, which is much higher than the MEV in Table 4, and obtained the values of *φ*_1x_, *φ*_1y_, and *φ*_1z_ as listed in Table 5. The divergence of the values of *φ*_1x_ is still obvious. And the values of *φ*_1z_ have lighter fluctuation than *φ*_1x_. Hence, we just give the ANOVA results of *φ*_1x_ in Table 6. At the level of *P* = 0.05, the significance of RN on *φ*_1x_ when *θ* = 0° is obvious. Especially, ANOVA fails when *θ* = 90°. The relationship between *φ*_1x_ and factors is not clear. Therefore, only mean value is valuable for experimentalists. In this case the mean value is 78.766° which is ~12° of deviation from 90°.

**Table 5:** Reflection angles of C60 with *v*_In_ = 20 Å/ps after impacting the nanobeam with different temperature. Units: reflection angle/°. (a) *θ* = 0°; (b) *θ* = 90°.

RN	T = 8 K			T = 100 K			T = 300 K			T = 500 K		
	*φ*_1x_	*φ*_1y_	*φ*_1z_	*φ*_1x_	*φ*_1y_	*φ*_1z_	*φ*_1x_	*φ*_1y_	*φ*_1z_	*φ*_1x_	*φ*_1y_	*φ*_1z_
**(a)**												
A1	−14.55	104.54°	90.56	−8.12	97.29	86.43	−18.88	108.38	94.17	−47.63	137.39	93.71
A2	12.33	77.69°	89.28	−1.72	90.20	88.29	25.06	64.94	89.78	48.28	41.85	92.68
A3	8.48	81.53°	89.62	−1.79	91.52	89.06	−19.08	108.43	85.26	−25.54	115.54	89.89
A4	6.06	83.98°	89.30	−5.36	94.45	87.03	−51.05	140.89	87.01	−42.48	132.49	90.35
A5	−15.75	105.73°	89.35	4.04	86.73	92.38	−6.30	93.10	84.56	−16.02	105.74	87.06
A6	−9.00	99.00°	90.29	−9.06	99.03	90.70	−12.94	102.41	93.63	−11.55	101.54	89.52
**(b)**												
A1	100.87	−10.92°	91.09	77.40	12.73	91.82	19.74	70.57	86.66	16.34	73.91	87.25
A2	76.00	14.23°	92.52	82.80	9.97	96.86	55.54	34.58	92.55	38.99	51.11	87.66
A3	79.70	10.35°	90.95	107.39	−17.38	89.99	143.28	−53.30	89.11	160.29	−72.67	80.89
A4	82.48	7.78°	91.98	93.39	−3.70	88.52	123.50	−33.51	90.72	−137.78	230.71	77.02
A5	105.07	−15.09°	90.69	86.25	3.79	89.13	84.74	5.44	91.37	106.18	−16.58	93.53
A6	93.01	−3.34°	91.45	97.91	−7.92	90.25	101.69	−12.05	88.65	95.52	−7.86	95.58

**Table 6:** ANOVA of *φ*_1x_. MV is the mean value of *φ*_1x_/(°).

*θ*	Source	DOF	SS	Mean square	*F* value	*P* value (*P*_r_ > *F*)	MV
0°	Model	8	6003.02	750.38	2.38	0.0700	−8.86
	RN	5	5123.58	1024.72	3.25	0.0346	
	T	3	879.44	293.14	0.93	0.4500	
	error	15	4723.10	314.87			
	Total	23	10726.11		*R*^2^ = 0.55966		
90°	Model	8	27852.14	3481.52	1.14	0.3931	78.76
	RN	5	19548.47	3909.69	1.28	0.3234	
	T	3	8303.67	2767.89	0.91	0.4614	
	error	15	45824.99	3055.00			
	Total	23	73677.12		*R*^2^ = 0.37803		

## Conclusions

For a resonator-based nanobalance, it can be used to measure the mass of a nanoparticle by comparing its natural frequencies with/without attracting the nanoparticle. Hence, the capability of capturing the nanoparticle during vibrating is essential for the nanobalance. By using a BNC nanotube with both clamped ends as a nanobalance and C60 as the nanoparticle, we estimate the capture capability of the nanobalance by both theoretical analysis and numerical experiments. According to the analysis and numerical results, some conclusions are drawn as following,

- Owing to the non-uniform surface of the BNC nanobeam, the nanoball escapes easier from the BN area of the beam surface than from the C area;
- Due to thermal vibration of the beam surface after collision, the MEV of nanoball can be more than one solution. If the colliding area on the beam surface moves with the nanoball along the same direction, the value of MEV is higher;
- The nanoball may slide for a few pica-seconds before being bounced out. Hence, the phonon propagation influence the escape moment and the value of MEV;
- The angle of reflection of the same incident nanoball has obvious divergence, in which indicates that the temperature-related velocity distribution on beam surface has serious effect on the reflection;
- At lower temperature, the MEV is higher, which means the capture capability of nanobalance is higher.
- To improve the capture capability of a nanobalance, the kinetic energy of the system, including the nanoparticle and the nanobeam, should be reduced as much as possible, and the van der Waals interaction needs to be improved if possible.

## Electronic supplementary material

Supplementary file 1

Supplementary file 1

Video 1

Video 2

Video 3

Video 4
