# Supplementary material for: Critical conditions for escape of a high-speed fullerene from a BNC nanobeam after collision
Source: Sci Rep. 2018 Jan 17;8:913. doi: 10.1038/s41598-017-18789-7 (PMC5772456; doi:10.1038/s41598-017-18789-7)
Supplement: Supplementary file 1 [file 41598_2017_18789_MOESM1_ESM.pdf]

Critical conditions for escape of a high-speed fullerene from a BNC nanobeam after collision

Kun Cai [1, 2*], Li-Kui Yang [1], Jiao Shi [1], Qing-Hua Qin [2*]

[1] *College of Water Resources and Architectural Engineering, Northwest A&F University, Yangling 712100, China*
[2] *Research School of Engineering, the Australian National University, ACT, 2601, Australia*
* Corresponding authors' Email: kuncai99@163.com (K. Cai); qinghua.qin@anu.edu.au (Q.H. Qin)

Supporting materials:

a) Movies:

Movie1—vIn=12, sita=30°, during [0, 14.5]ps.avi;
Movie2—vIn=7.01, sita=90°, during [1.35, 2.87]ps.avi;
Movie3—A1-B2, sita=60° during [0, 15.5]ps.avi;
Movie4—A6-B1, sita=60° during [0, 19.5]ps.avi.
