# Supplementary material for: Critical conditions for escape of a high-speed fullerene from a BNC nanobeam after collision
Source: Sci Rep. 2018 Jan 17;8:913. doi: 10.1038/s41598-017-18789-7 (PMC5772456; doi:10.1038/s41598-017-18789-7)
Supplement: Supplementary file 2 — Supplementary file 1 [file 41598_2017_18789_MOESM2_ESM.pdf]

Critical conditions for escape of a high-speed fullerene from a BNC nanobeam after collision

Kun Cai [1, 2*], Li-Kui Yang [1], Jiao Shi [1], Qing-Hua Qin [2*]

[1] *College of Water Resources and Architectural Engineering, Northwest A&F University, Yangling 712100, China*

[2] *Research School of Engineering, the Australian National University, ACT, 2601, Australia*

* Corresponding authors' Email: kuncai99@163.com (K. Cai); qinghua.qin@anu.edu.au (Q.H. Qin)

Supporting materials

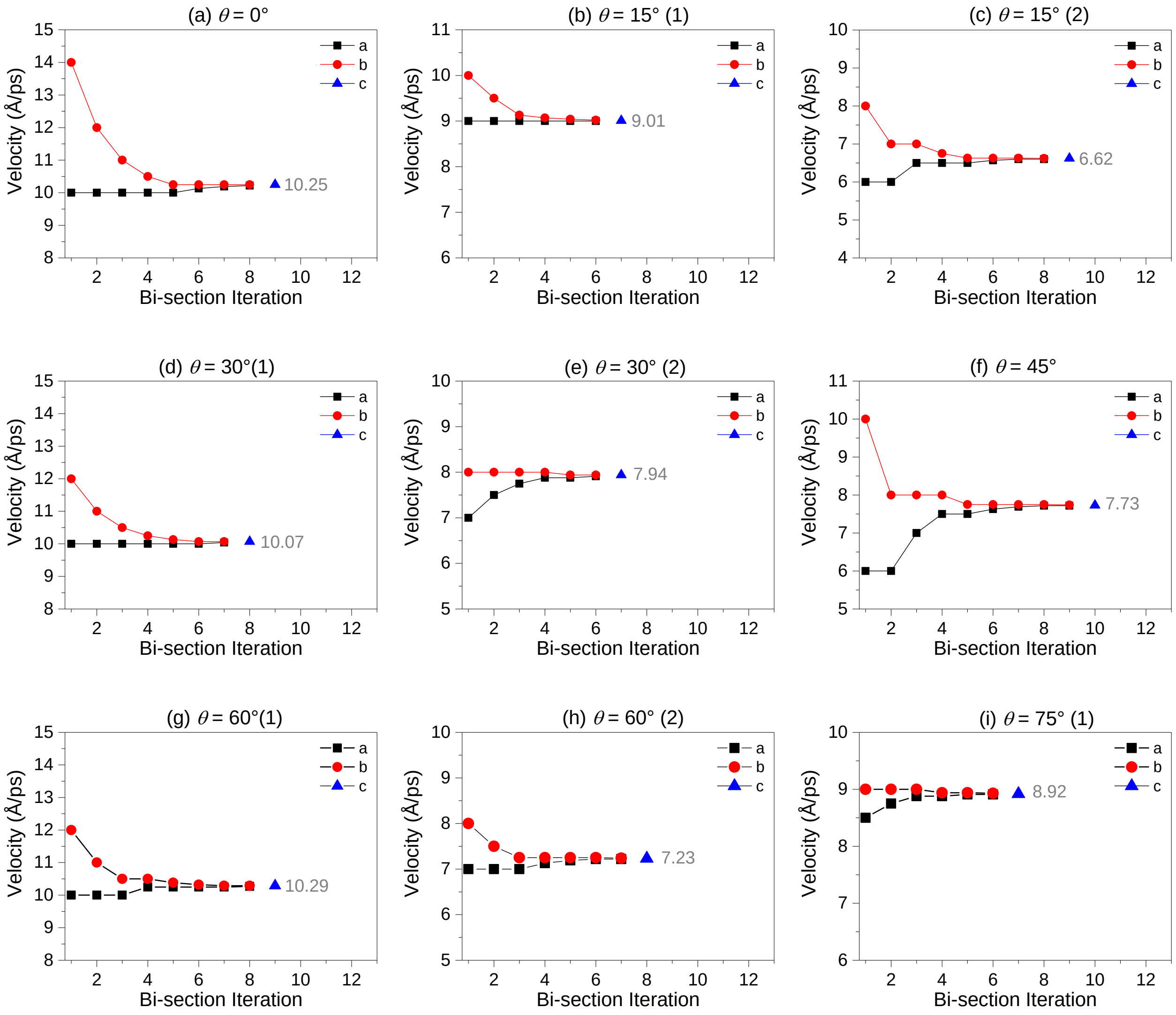

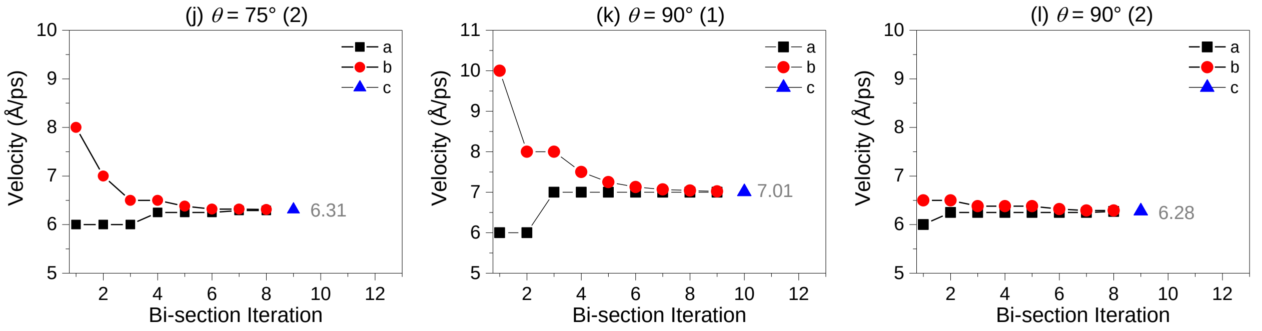

S-Figure 1 Process of bi-section algorithm for finding the critical value of escape velocity of C60 after impact BNC nanobeam from different direction at 8K.

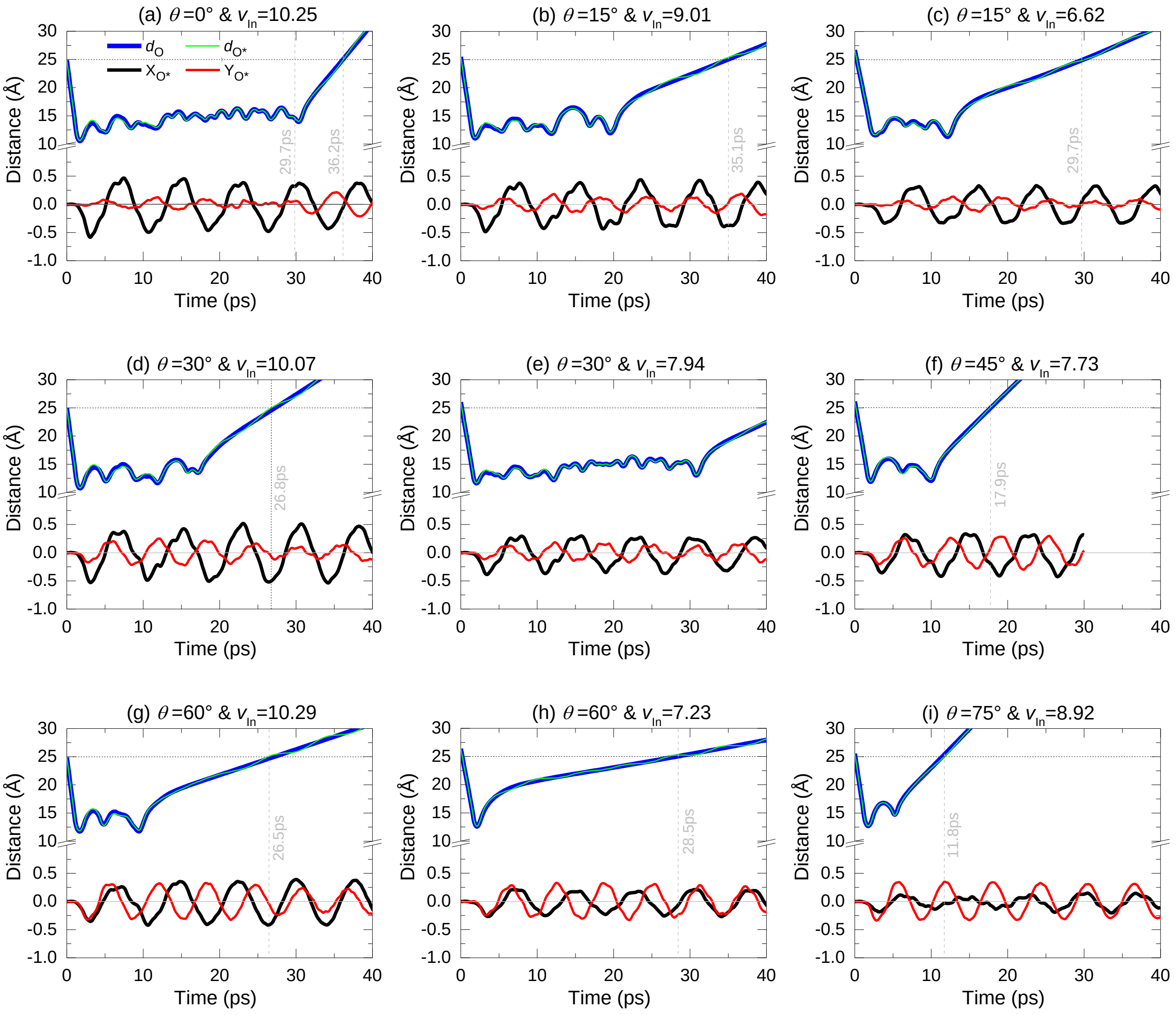

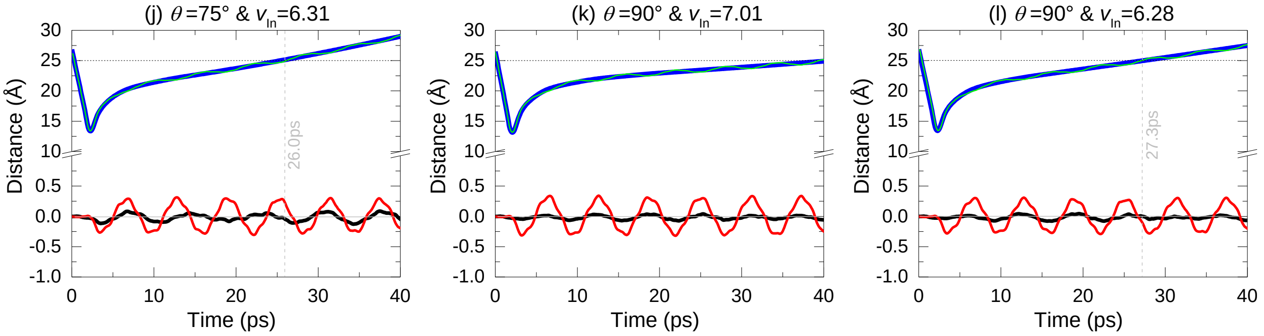

S-Figure 2 Histories of the distances between the mass center of C60 and origin O (blue curve), or between the mass center of nanobeam (orange curve), and the positions of mass center of nanobeam (X_{O*} in black curve, Y_{O*} in red curve).
